# Structural effects on the catalytic activity of carbon-supported magnetite nanocomposites in heterogeneous Fenton-like reactions†

**DOI:** 10.1039/c8ra02286k

**Published:** 2018-05-01

**Authors:** Hongmei Zang, Chunyan Miao, Jianying Shang, Yingxin Liu, Juan Liu

**Affiliations:** The Key Laboratory of Water and Sediment Sciences, Ministry of Education, College of Environmental Sciences and Engineering, Peking University Beijing 100871 China juan.liu@pku.edu.cn; School of Gemmology, China University of Geosciences Beijing, 100083 China; Department of Soil and Water Sciences, China Agricultural University Beijing 100193 China

## Abstract

The catalytic reactivity of synthetic bare magnetite nanoparticles, activated carbon supported magnetite (AC-Mt), and graphene oxide supported magnetite (GO-Mt) for heterogeneous Fenton-like oxidation of methylene blue (MB) were compared, in order to investigate how the structural features of the support impact catalytic activity of the nanocomposites. The different effects of AC and GO on MB removal rate, hydroxyl radical (˙OH) production, iron leaching, and surface deactivation have been systematically studied. The rate constant of MB removal by AC-Mt was 0.1161 min^−1^, one order of magnitude larger than the value of bare magnetite nanoparticles (0.0566 min^−1^). The higher catalytic activity of AC-Mt might be attributed to the larger reactive surface area of well-dispersed magnetite for ˙OH production and the recharge of the magnetite surface by the AC support *via* Fe–O–C bonds. However, the removal rate of MB by GO-Mt was one order of magnitude slower than that of bare magnetite nanoparticles under the same experimental conditions, presumably due to the wrapping of GO around magnetite nanoparticles or extensive aggregation of GO-Mt composites. These findings revealed the significant influence of support structure on the catalytic activity of carbon-supported magnetite nanocomposites, which is important for the development of efficient magnetite-based catalysts for wastewater treatments.

## Abstract 2

This study revealed that the geometrical structure of a carbonaceous support can significantly impact the catalytic activity of the whole nanocomposites.

## Graphical abstract

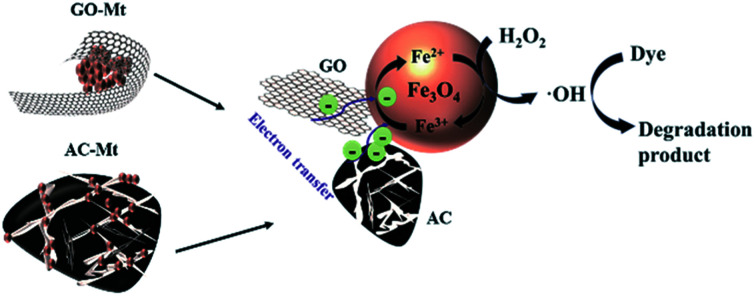

## 1. Introduction

Heterogeneous Fenton-like reaction is one of the most promising advanced oxidation processes (AOPs) that can produce highly reactive hydroxyl radicals (˙OH) to non-selectively oxidize organic and inorganic pollutants^1^ and has many advantages over the conventional homogeneous Fenton process, such as long term stability of catalysts and no iron sludge formation.^1–4^ However, it is critical to understand the mechanisms of the heterogeneous Fenton reaction in order to develop its applications in wastewater treatments.

Magnetite, a widespread iron oxide mineral in natural sediments,^5^ is a promising heterogeneous Fenton-type catalyst for wastewater treatment because of its good biocompatibility and unique ferromagnetism. The mixed-valence states for iron ions in magnetite structure may facilitate the initiation and propagation stages of Fenton processes, making magnetite a good catalyst for heterogeneous Fenton-like reactions. Nevertheless, surface oxidation of magnetite during Fenton reactions may lead to a gradual decrease in catalytic reactivity.^6,7^ Moreover, magnetite particles tend to aggregate in aquatic environments, which may evidently reduce available surface active sites for catalysis.^8^ Thus, immobilization of magnetite particles onto high-surface-area supports, such as mesoporous silica, clay minerals, activated carbon, zeolites, *etc.*, has been proposed as an environmentally-friendly solution to these problems.^4^

Among all substrates used for magnetite-based catalysts in heterogeneous Fenton reactions, carbonaceous materials have aroused wide interest because of their high specific surface area, stability in acidic/basic media, tunable surface chemistry and structure, as well as environmental compatibility.^9–11^ Carbonaceous materials with different morphologies, such as one-dimensional carbon nanotubes, two-dimensional graphene sheets, and three-dimensional activated carbon with high porosity, have been proposed as the support for magnetite-based catalysts.^12^ The reactivity of these nanocomposites as heterogeneous Fenton catalysts has been widely studied in the treatments of various contaminants,^9^ but most of the studies focused on the interrelationship between chemical properties of carbon materials (such as metal impurities, surface functional groups, *etc.*) and catalytic efficiency of the carbon-magnetite nanocomposites. Very limited studies have studied how structural features of carbonaceous materials affect the catalytic activity of the whole nanocomposites. A recent study found that the ratio of magnetite to graphene oxide in graphene oxide-Fe_3_O_4_ nanocomposites led to different nanocomposite structures and catalytic activities for the heterogeneous Fenton-like reaction.^12^ Magnetite nanoparticles (Mt NPs) were dispersed on GO nanosheets at low GO loading (5 wt%), while Mt NPs were covered by GO stacking in the nanocomposite with higher GO loading (15 wt%).^12^ Moreover, Zhou *et al.* reported that, in the synthetic graphene-Mt composites with a 13.3 wt% graphene content, the graphene nanosheets enwrap Mt NPs.^13^ Further studies are needed to investigate the relationships between nanocomposite structure and their catalytic activity for development of highly efficient Fenton-like catalyst.

In order to investigate how substrate structure influences the catalytic activity of the carbon-supported nanocomposites, in this study, we selected graphene oxide as the representative two-dimensional lamellar support and activated carbon as the typical three-dimensional porous support to prepare carbon-supported magnetite nanocomposites. We synthesized activated carbon supported magnetite (AC-Mt) and graphene oxide supported magnetite (GO-Mt) nanocomposites with the same magnetite-to-substrate ratio of 1 : 1, and compared the catalytic reactivity of synthetic bare magnetite nanoparticles, AC-Mt, and GO-Mt in heterogeneous Fenton-like oxidation of methylene blue (MB), a model recalcitrant dye pollutant. Moreover, aggregation state, adsorption capacity for MB, Fe leaching, ˙OH production, changes in surface chemistry of these catalysts have been systematically studied. The findings in the present study revealed that the geometrical structure of carbonaceous support can significantly impact the dispersion, available surface sites, Fe leaching, and surface oxidation of the supported magnetite nanoparticles, leading to obviously different catalytic activities in heterogeneous Fenton reactions.

## 2. Experimental

### 2.1. Synthesis of GO, Mt, GO-Mt, and AC-Mt

The materials used in this paper were shown in the ESI Section S1.† GO was synthesized from graphite powder using a modified Hummers method.^14^ Mt nanoparticles (NPs) were synthesized in an anoxic glovebox (N_2_ atmosphere, lower than 1 ppm residual O_2_) according to the co-precipitation method.^15^ The GO-Mt or AC-Mt nanocomposites were synthesized in the anoxic glovebox similarly *via* the chemical co-precipitation method described above, but with the addition a certain amount of AC and GO, representatively, to obtain the magnetite-to-substrate ratio of 1 : 1. The details of the synthesis of GO, Mt, GO-Mt and AC-Mt can be found in Section S2.†

### 2.2. Characterization of synthetic nanomaterials

The crystalline phase of synthetic materials was characterized by powder X-ray diffraction using a Rigaku D/MAX-2000 diffractometer with monochromatic CuKα radiation (*λ* = 0.15406 nm) at a scan rate of 0.02 2*θ* s^−1^. Freeze-dried samples were loaded on quartz slides for XRD measurements. The morphology of the synthetic catalysts was observed on a cold field-emission scanning electron microscope (SEM, Hitachi, SU8020) at an accelerating voltage of 5.0 kV in secondary electron imaging (SEI) mode. Energy Dispersive X-ray Spectroscopy (EDS, TEAM™ EDAX Analysis system) was used to analyze the elemental composition of the nanocomposites at an accelerating voltage of 5.0 kV and a take-off angle of 34.4 degree. The primary particle diameters of synthetic Mt NPs were examined with a field-emission transmission electron microscope (TEM, JEOL JEM-2100F). Brunauer–Emmett–Teller (BET) specific surface area was measured using a Micrometer ASAP2020. The mass ratios of magnetite to support in nanocomposites were determined by acid digestion and particle concentration measurements (Section S2†).

The change of surface chemical properties of Mt, AC-Mt, and GO-Mt before and after Fenton reactions were analyzed by X-ray photoelectron spectroscopy (XPS). All XPS spectra were recorded with a Kratos AXIS-Ultra spectrometer (Kratos Analytical, Manchester, UK) employing a monochromatic Al K_α_ radiation (150 W) and a low-energy electron flooding for charge compensation. The carbon 1s line at 284.6 eV (for hydrocarbon or hydrocarbon groups) was used to calibrate the binding-energy scale for XPS measurements.

### 2.3. Adsorption kinetics of MB on catalysts

The removal of MB by bare Mt and supported Mt involve both the adsorption of MB on the surface of catalysts and Fenton oxidation of MB. To evaluate the contribution of adsorption to the removal of MB by the synthetic materials, the adsorption kinetics of 10 mg L^−1^ MB on the synthetic Mt NPs, GO-Mt, and AC-Mt, as well as pristine AC and GO, were conducted in a batch reactor at room temperature. The initial particle concentration was 13 mg L^−1^ in all adsorption experiments. In addition, MB adsorption on 6.5 mg L^−1^ pristine AC or GO was also studied, considering GO or AC only accounted for 50% of the weight in GO-Mt and AC-Mt nanocomposites. A certain volume of the stock suspension was added to 100 mL degassed HCl solution (pH = 3) in a 150 mL polytetrafluoroethylene flask and mixed for 20 min in the anaerobic glovebox. 667 μL of MB stock solution (1.5 g L^−1^) was spiked into the reactor to initiate the adsorption experiment. At predetermined time intervals, 3 mL sample was taken and filtered through 0.22 μm syringe filters. The MB concentration in the filtrate was immediately determined using an UV-Vis spectrophotometer (UV-1800, SHIMADZU) by measuring the absorbance at 665 nm. All experiments were conducted in triplicates under constant stirring (350 rpm) at room temperature and in the dark.

### 2.4. Heterogeneous Fenton-like degradation of MB

The experimental procedures and conditions of the heterogeneous Fenton-like degradation experiments were similar to those for the adsorption experiments described above, but 21 μL H_2_O_2_ (30%) was added simultaneously with the MB stock solution in order to initiate Fenton-like reactions.^16^ The total organic carbon (TOC) content of MB solution before and after 4 hours heterogenous Fenton oxidation were determined by a TOC meter (TOC-VCPN, Shimadzu, Japan) to evaluate the mineralization of MB. In addition, reusability of the synthetic catalysts under the same conditions was studied in seven consecutive cycles of use. The catalyst after each cycle was separated magnetically from suspensions and then washed three times with degassed and deionized Milli-Q water (DDW) before being added into a fresh MB solution. Because the loss of catalysts during recycling processes was inevitable, the initial weight concentration of catalysts in the seven-cycle experiments was increased to 65 mg L^−1^ in order to minimize the influence of catalyst loss. Other experimental conditions are kept the same.

In order to compare the ability of different catalysts to generate hydroxyl radical (˙OH) in the Fenton-like reactions, ˙OH was quantified according to the method reported in the previous study.^17^ More details were shown in Section S3.† Furthermore, to study the role of ˙OH in MB removal, parallel experiments of Fenton-like reactions were conducted with the addition of 100 mM methanol as a hydroxyl radical scavenger.^18^ The MB concentrations in the system were monitored over time to evaluate the effect of ˙OH scavenging on MB removal.

The aggregation state of Mt NPs, GO and GO-Mt, respectively, at 25 °C under the similar solution conditions for the Fenton experiments were investigated by dynamic light scattering (DLS) on a Zetasizer (Nano ZS90, Malvern, UK) operating with a He–Ne laser at a wavelength of 633 nm and a scattering angle of 90°. A certain volume of the stock suspensions was added to 5 mL degassed HCl solution (pH = 3) in 10 mL centrifuge tubes. The mixture were sonicated (40 kHz, 300 W) for 20 min just before measurements. All measurements were conducted in three runs with at least fifty measurements for each run.

## 3. Results and discussion

### 3.1. Characterization of synthetic catalysts

**Fig. 1:**
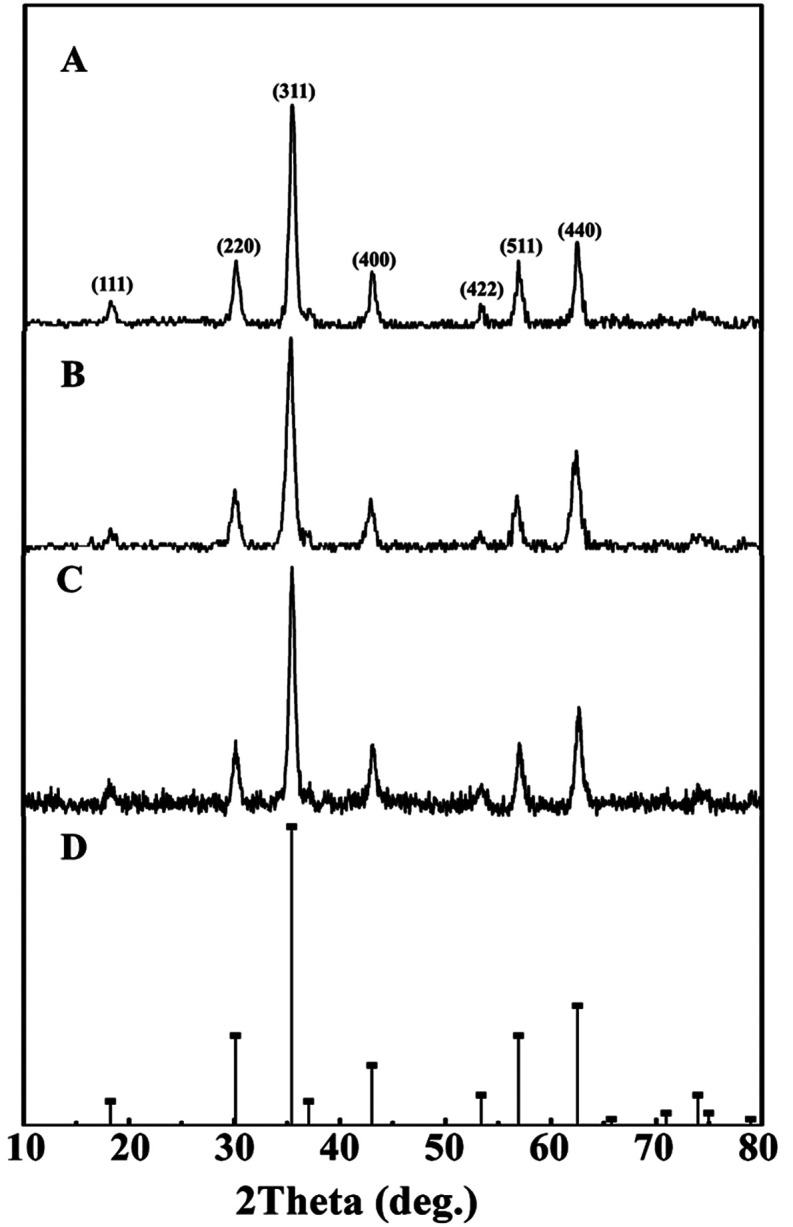
XRD patterns of synthetic Mt (A), AC-Mt (B), and GO-Mt NPs (C) with the reference pattern of magnetite (PDF no. 19-0629) (D).

XRD patterns of synthetic bare Mt NPs, AC-Mt, and GO-Mt (Fig. 1) indicated that only magnetite was present in all samples. Although pristine activated carbon shows a broad and weak peak around 2*θ* = 15–30° (Fig. S1A†), the signal of the AC substrate in AC-Mt was too weak to be observed in its XRD pattern. The similar results been previously reported in the XRD patterns of activated carbon-supported composites.^19,20^ The lack of the GO peak at 2*θ* = 11.2° (Fig. S1B†) in GO-Mt is consistent with the previously reported results of GO-Fe_3_O_4_ composites, which is probably due to the destruction of regular stacking of GO sheets by the decoration of Mt NPs.^21,22^

**Fig. 2:**
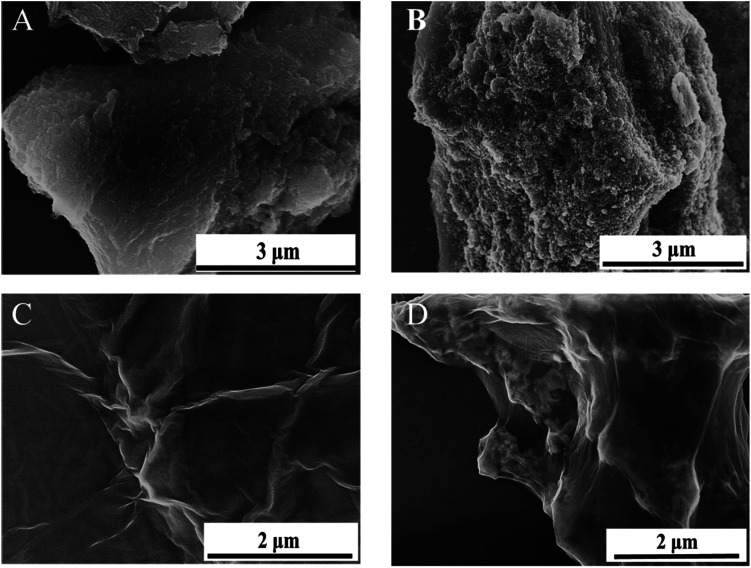
SEM images of pristine AC (A), AC-Mt (B), GO (C), and GO-Mt (D).

The synthetic Mt NPs were nearly spherical with a diameter of ∼11 nm (Fig. S2†). The representative SEM images of the pristine AC, GO, AC-Mt, and GO-Mt are shown in Fig. 2. Compared to pristine AC (Fig. 2A), AC-Mt composite showed a much rougher surface with brighter aggregates all over the sample (Fig. 2B), indicating the well-dispersion of magnetite NP aggregates on the surface or in the holes of AC. The EDS spectrum of AC-Mt (Fig. S3†) indicated that the chemical composition of the bright aggregates on AC surface included Fe, O, and C, indicating the formation of Mt NPs.

The synthetic GO exhibited a sheet-like morphology with large lateral surface and wrinkled edge (Fig. 2C), while GO-Mt (Fig. 2D) showed the wrapping of magnetite NPs by GO nanosheets. A similar phenomenon that GO nanosheets fold and wrapped goethite (FeO(OH)) NPs was observed after mixing GO and goethite suspensions.^23^ The possible explanation is that the GO nanosheets are not thick enough to stably support NPs and apt to fold around NPs or form crimps at the edge of NPs. In addition, to synthesize the GO-Mt nanocomposites with the same magnetite-to-substrate ratio (1 : 1) as AC-Mt, a relatively high GO loading was used during the synthesis, which inevitably led to the wrapping of GO around Mt NPs.^12^ The SEM images of AC-Mt and GO-Mt (Fig. 2) directly show that using AC or GO as the support for carbon-supported magnetite catalysts could lead to the different structures of nanocomposites. The wet chemical analysis (Section S2†) confirmed that the mass ratio of magnetite to support in GO-Mt and AC-Mt was about 1 : 1.

### 3.2. MB adsorption on catalysts

**Fig. 3:**
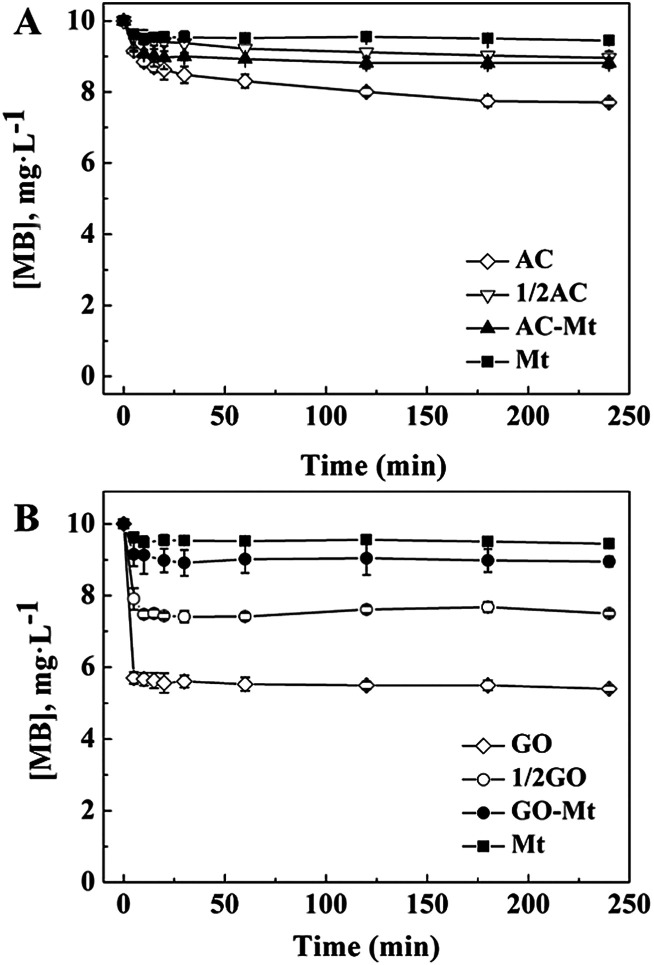
(A) Comparison of MB adsorption on 13 mg L^−1^ bare Mt, pristine AC, and AC-Mt; (B) the different adsorption capacity between 13 mg L^−1^ bare Mt, pristine GO, and GO-Mt; 1/2 AC in (A) and 1/2 GO in (B) indicate the amount of added adsorbents was 6.5 mg L^−1^.

The adsorption of MB on bare Mt NPs, AC-Mt, and GO-Mt, as well as pristine AC and GO, were, respectively, measured under the similar experimental conditions for heterogeneous Fenton-like reactions but without H_2_O_2_ (Fig. 3). At equilibrium, only 5.0% of 10 mg L^−1^ MB was adsorbed on 13 mg L^−1^ bare Mt NPs. The low adsorption capacity of Mt NPs for MB was consistent with previous results.^24^ About 10.1% and 11.8% of MB were adsorbed on 13 mg L^−1^ GO-Mt and AC-Mt, respectively. The results suggest that either bare Mt or supported Mt nanocomposites only adsorbed a small portion of 10 mg L^−1^ MB. The observed difference in MB removal by these synthetic materials was not simply attributed to the higher adsorption capacity of the supports.

It is worth mentioning that the adsorption capacity of 13 mg L^−1^ AC-Mt (AC : Mt = 1 : 1) was similar to that of 6.5 mg L^−1^ pristine AC (1/2 AC in Fig. 3A), indicating that MB was mainly adsorbed by the AC support in the case of AC-Mt. In addition, the specific surface area (Table S1†) of AC (962.0 m^2^ g^−1^) was almost twice of AC-Mt (512.1 m^2^ g^−1^), which agreed with the similar adsorption capacity of 6.5 mg L^−1^ pristine AC and 13 mg L^−1^ AC-Mt, as shown in Fig. 3A.

However, the adsorption capacity of 13 mg L^−1^ GO-Mt (GO : Mt = 1 : 1) was much lower than 6.5 mg L^−1^ (1/2 GO in Fig. 3B) or 13 mg L^−1^ pristine GO. The lower adsorption capacity of GO-Mt than pristine GO might be related to the extensive aggregation of GO-Mt that resulted in the decrease of reactive sites for MB adsorption. The hydrodynamic diameters (*D*_h_) of 13 mg L^−1^ GO-Mt in the suspension at pH 3 was 1677 ± 95 nm, evidently larger than the *D*_h_ of 13 mg L^−1^ pristine GO (833 ± 212 nm) or bare Mt NPs (116 ± 3 nm). Mt and GO are opposite charged at pH 3. Extensive aggregation of GO-Mt can be induced *via* electrostatic attraction between Mt and exposed GO surface,^25^ resulting in the decrease of surface sites available for MB adsorption.

MB removal by pristine AC or GO in the presence of H_2_O_2_ (data not shown) were similar to their corresponding adsorption curves of MB (Fig. 3), so the support in AC-Mt or GO-Mt could not catalyze Fenton-like oxidation of MB by themselves under the conditions of this study.

### 3.3. Fe-leaching induced homogeneous Fenton reactions

**Fig. 4:**
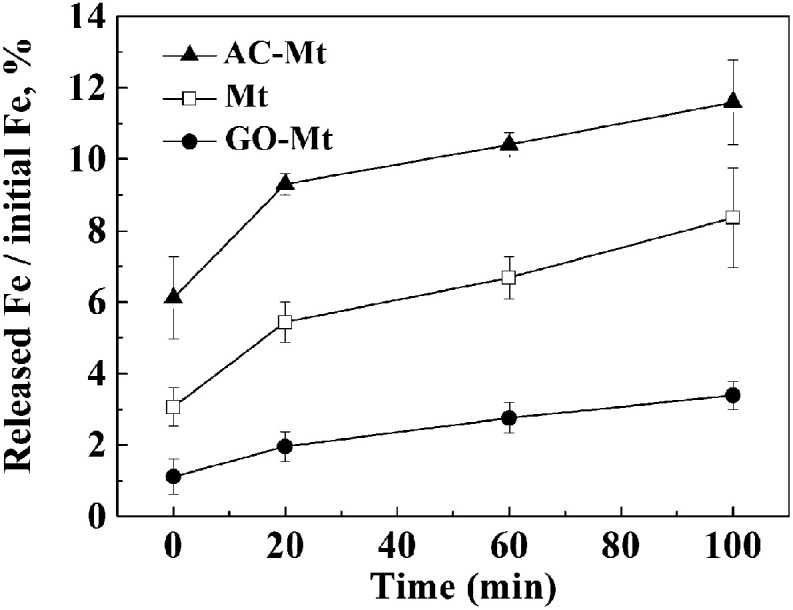
The percentage of Fe released from 13 mg L^−1^ catalysts as a function of time in the solution for heterogeneous Fenton reactions.

In the solutions for Fenton process at pH 3, magnetite may release iron ions due to proton-promoted dissolution of magnetite and consequently induce homogeneous Fenton reactions.^1,9^ To study the effect of different supports on Fe leaching from the synthetic catalysts, the percentage of Fe released from 13 mg L^−1^ catalysts as a function of time under the conditions similar to those of heterogeneous Fenton-like experiments were compared in Fig. 4. When 13 mg L^−1^ catalyst was added, 9.3%, 2.0%, and 5.4% of total Fe was, respectively, released from AC-Mt, GO-Mt and bare Mt after 20 minutes. Although the initial Fe concentration in bare Mt NPs was two times higher than that of GO-Mt or AC-Mt, the percentage of Fe leaching in AC-Mt was still much higher than the value of bare Mt. The three-dimensional porous structure of activated carbon allowed magnetite NPs to be well dispersed, effectively inhibited aggregation of Mt NPs, and therefore promoted proton-promoted dissolution of Mt NPs. On the contrary, the percentage of Fe released from GO-Mt was obviously less than the values from the other two catalysts. The inhibition of magnetite dissolution by GO nanosheets was probably due to the wrapping of GO around magnetite NPs or extensive aggregation of GO-Mt composites (Fig. 2D). Likewise, graphene has been proposed as a corrosion-protecting coating to decrease corrosion rates of metallic surfaces.^26,27^ Thus, the support with different structure can significantly influence the dispersion and accessible reactive surface area of Mt NPs in the supported magnetite nanocomposites.

In all heterogeneous Fenton-like experiments, before MB and H_2_O_2_ were added, catalysts were pre-equilibrated with the solution at pH 3 for 20 minutes, because the rates of Fe leaching from these catalysts obviously decreased after 20 minutes. Fig. 4 shows that 0.51, 0.44, and 0.09 mg L^−1^ Fe ions were, respectively, released from bare Mt NPs, AC-Mt, and GO-Mt at 20 minutes. Previous studies have proven that the dissolved iron ions mostly are Fe^3+^ in heterogeneous Fenton-like system with magnetite at pH = 3,^28,29^ so Fenton oxidation of 10 mg L^−1^ MB with 0.44, 0.51, and 0.09 mg L^−1^ Fe^3+^ ions, respectively, were studied in order to evaluate the contribution of Fe-leaching induced homogeneous Fenton reaction to MB removal by the different catalysts. As shown in Table 1, the higher Fe^3+^ concentration resulted in the higher oxidation rates of MB. Thus, the rate constants of MB removal by iron ions released from bare Mt, AC-Mt, and GO-Mt were 0.0114, 0.0078, and 0.0023 min^−1^, respectively.

**Table 1:** The apparent pseudo-first-order rate constants (*k*_app_) of MB removal and the generation rates of ˙OH in the Fenton oxidation of MB with different catalysts

Catalysts	Heterogeneous Fenton		˙OH production		Iron leaching	Homogeneous Fenton
	*k*_app_ (min^−1^) 1^st^ stage	*k*_app_ (min^−1^) 2^nd^ stage	Within 5 min (μM min^−1^)	Within 25 min (μM min^−1^)	mg L^−1^	*k*_app_ (min^−1^)
AC-Mt	0.1161		3.58	0.83	0.44	0.0078
Mt	0.0566		2.27	0.45	0.51	0.0114
GO-Mt	0.0075	0.0282	1.1	0.46	0.09	0.0023

### 3.4. Heterogeneous Fenton-like reaction

**Fig. 5:**
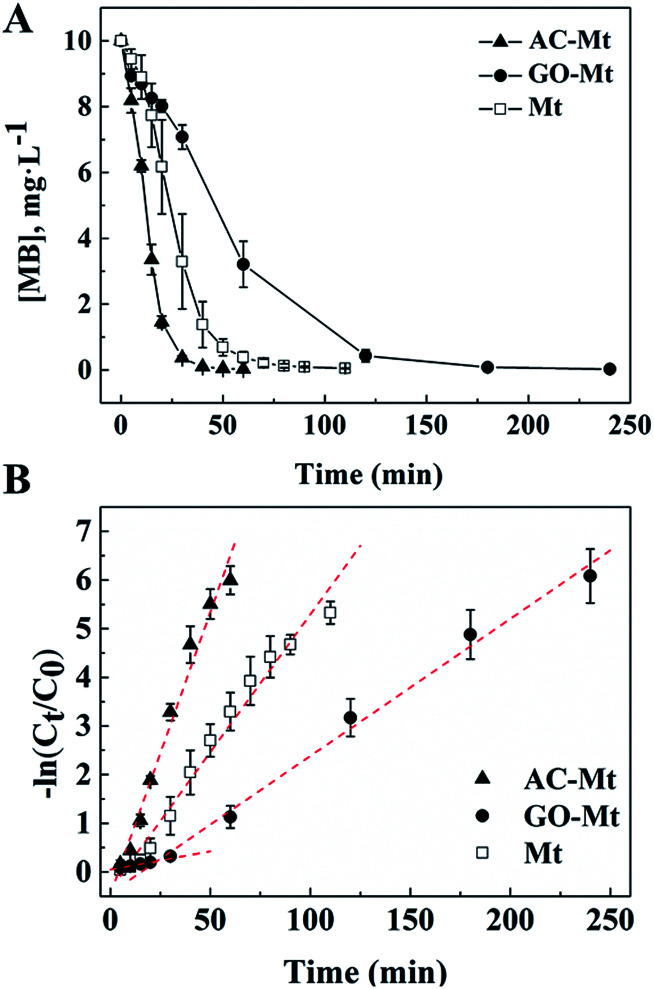
(A) Concentrations of MB as a function of time in heterogeneous Fenton-like reactions with AC-Mt, GO-Mt and bare Mt, respectively; (B) first-order polynomial fit to the kinetic data (A) of MB removal by AC-Mt, GO-Mt, and bare Mt, respectively.

Kinetics of MB removal in the presence of 13 mg L^−1^ bare Mt NPs, AC-Mt, and GO-Mt, respectively, were shown in Fig. 5. To quantitatively compare the catalytic activity of these catalysts, the pseudo-first-order model was used to calculate the initial degradation rates of MB:^16,30^

1 −ln(*C*_0_/*C*_*t*_) = *k*_app_*t*

where *C*_0_ and *C*_*t*_ are the concentrations of MB (mM) at the initial time and the reaction time *t*, respectively. *k*_app_ is the apparent pseudo-first-order rate constant (min^−1^), and *t* is the degradation time (min). In all cases, the degradation data of MB in the initial stage (within the first 20 min) fitted the kinetic model (eqn (1)) very well with a *R*^2^ greater than 0.98. The initial rate constants of MB removal with the three catalysts were in the order: AC-Mt (0.1161 min^−1^) > Mt (0.0566 min^−1^) > GO-Mt (0.0075 min^−1^) (Table 1). The results show that the use of AC as the support efficiently increased the rate constant of MB removal by an order of magnitude, compared to bare Mt NPs. On the contrary, when GO was used in the nanocomposite, the initial removal rate of MB decreased one order of magnitude under the same experimental conditions.

Because the mass ratio of magnetite to support in the synthetic GO-Mt or AC-Mt composites was 1 : 1, control experiments with 6.5 mg L^−1^ bare Mt was conducted to investigate the effect of initial Fe_3_O_4_ concentration on the kinetics of MB removal. The initial rate constant of MB removal with 6.5 mg L^−1^ bare Mt was 0.0511 min^−1^ (*R*^2^ = 0.95) that was very close to the value (0.0566 min^−1^) of 13 mg L^−1^ bare Mt (Table 1). Thus, the different removal rates of MB in the experiments with bare Mt and the supported Mt nanocomposites were not mainly due to the different amount of magnetite in the catalysts.

**Fig. 6:**
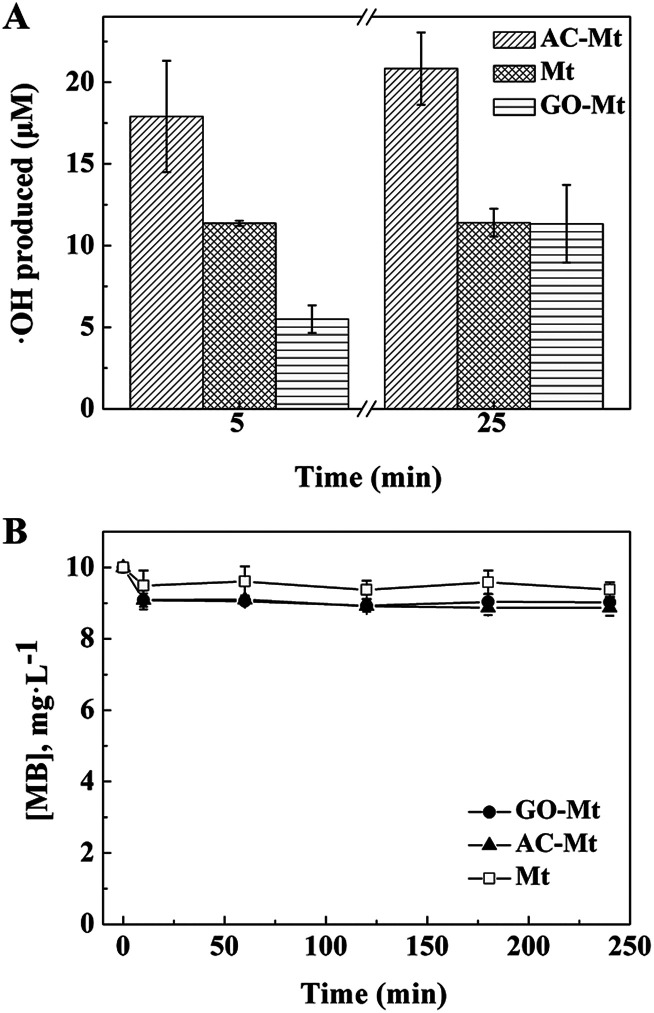
(A) The concentration of ˙OH produced in heterogeneous Fenton-like systems with different catalysts. (B) Concentrations of MB as a function of time in heterogeneous Fenton-like reactions in the presence of methanol as the ˙OH scavenger.

The initial rates of heterogeneous Fenton-like reactions with any of the synthetic catalysts were evidently larger than the corresponding rates of the homogeneous Fenton reactions induced by released iron ions (Table 1). For example, the rate constant of MB removal by 13 mg L^−1^ AC-Mt was 0.1161 min^−1^ that was two orders of magnitude faster than the rate constant (0.0078 min^−1^) of the homogeneous Fenton oxidation by 0.44 mg L^−1^ iron ions released from 13 mg L^−1^ AC-Mt. Moreover, although bare Mt released more iron ions than AC-Mt, the initial rate of MB removal by AC-Mt was obviously higher than bare Mt. Thus, MB removal by these catalysts was mainly attributed to heterogeneous Fenton-like process, not Fe-leaching induced homogeneous Fenton process. The smaller initial rate of MB removal by GO-Mt was probably related to the wrapping of magnetite NPs by GO nanosheets or extensive aggregation of GO-Mt nanocomposites, which inhibited the interaction between magnetite and H_2_O_2_ or the diffusion of reactive oxygen species and MB. As shown in Fig. 5 and Table 1, the rate constant of MB removal by GO-Mt increased to 0.0282 min^−1^ after ∼20 minutes, showing a two-stage reaction. It might be related to the continuous increase of iron ions released from GO-Mt or ˙OH concentration after 20 minutes (Fig. 4 and 6A).

The initial TOC content in MB solution was 5.9 mg L^−1^. After 4 hours heterogeneous Fenton oxidation, the TOC concent in the systems with AC-Mt, GO-Mt and Mt was 1.9, 2.1 and 2.4 mg L^−1^, respectively. As shown in Fig. 5A, MB was completely removed after 4 hours reactions in all cases. However, the TOC removal efficiency by AC-Mt, GO-Mt and Mt was 68%, 64% and 59%, respectively, which were consistent with the previously reported results about MB removal by magnetite-based catalysts *via* heterogeneous Fenton reaction.^31–33^ The residual TOC may be attributed to some small molecular intermediates generated from the catalytic reactions.^31^

The reusability of bare Mt, AC-Mt, and GO-Mt was studied, respectively, in seven consecutive cycles of use. The percentage of MB removal after seven successive cycles by all catalysts was still more than 85% of MB, indicating a good recyclability of all catalysts for the heterogeneous Fenton-like oxidation of MB. The removal efficiency of MB during the first four hours in each cycle was compared in Fig. S4.† The gradually decrease of initial oxidation rates, or catalytic efficiency, with the increase of cycles was observed in all cases. It might be related to Fe leaching from magnetite, the oxidation of magnetite surface, or poisoning of the active catalytic sites by the adsorbed organic species.^29,34,35^

### 3.5. Effects of different supports on ˙OH generation

In Fenton oxidation reactions, MB is mainly oxidized by hydroxyl radicals that are generated by catalysts in H_2_O_2_.^36^ To further investigate how the support affected the catalytic activity of composites, the concentrations of ˙OH produced by bare Mt, AC-Mt, and GO-Mt were compared in Fig. 6A. In all cases, the generation rate of ˙OH was relatively faster in the first 5 minutes and then slowed down with time. The trend agrees well with previously reported results.^17^ When the same mass concentration of catalysts was used, the concentrations of ˙OH produced by different catalysts at 5 min were in the order: AC-Mt > Mt > GO-Mt, which agreed well with the order of MB removal rates (Table 1). The relatively lower rate of ˙OH production by GO-Mt can be attributed to the wrapping of GO on magnetite NPs or extensive aggregation of GO-Mt that inhibited the interaction between H_2_O_2_ and magnetite surface. From 5 to 25 minutes, the generation rate of ˙OH by bare Mt was negligible probably due to surface oxidation or deactivation,^1,29^ while the ˙OH concentration of GO-Mt increased to the value similar to that of bare Mt. Thus, the GO support inhibited the production of ˙OH by GO-Mt, but also alleviated surface deactivation of magnetite.

The concentration of hydroxyl radicals in the system with AC-Mt was 17.9 μM at 5 minutes and increased to 20.8 μM at 25 minutes (Fig. 6A) that were obviously higher than the values of bare Mt and GO-Mt. In AC-Mt, Mt NPs were well-dispersed in AC that has a large specific surface area and porous structure. Relative to aggregated Mt NPs in solution, AC-Mt has more active surface sites for the reaction with H_2_O_2_, resulting in more ˙OH production. Besides, methanol was used as a scavenger for ˙OH during heterogeneous Fenton-like experiments (Fig. 6B). The degradation profiles of MB by the catalysts became similar to the corresponding adsorption curves (Fig. 3). Thus, ˙OH was the predominant reactive species to oxidize MB in the heterogeneous Fenton-like reaction with bare Mt or Mt-based nanocomposites.

### 3.6. Changes in surface properties of catalysts

**Fig. 7:**
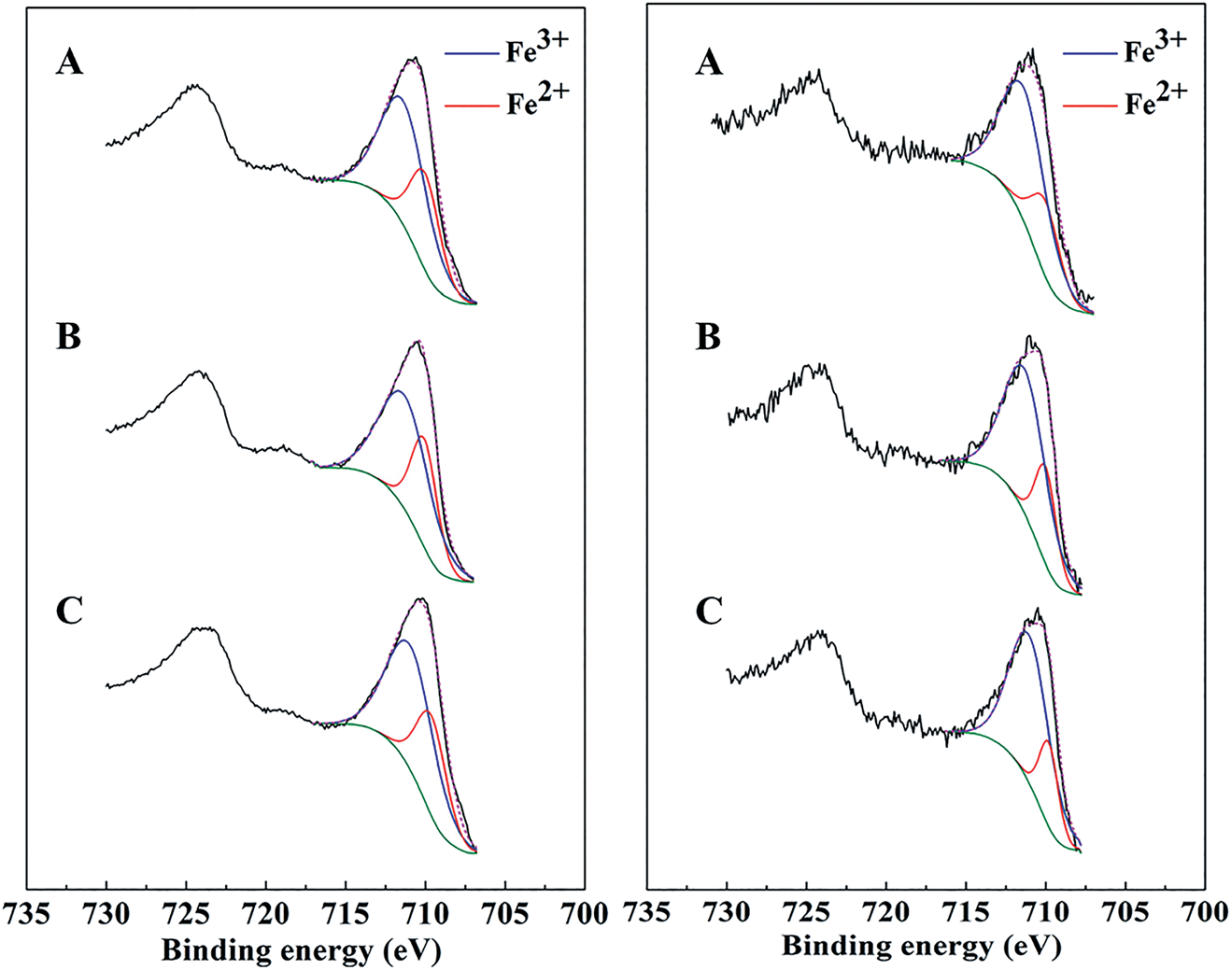
XPS Fe 2p peaks of AC-Mt (A), GO-Mt (B), and Mt (C) before (left) and after (right) seven consecutive cycles of use.

XPS spectra of bare Mt, AC-Mt, and GO-Mt before and after seven consecutive cycles were compared in Fig. 7. The Fe 2p_3/2_ spectrum of all catalysts before Fenton reactions was composed of two peaks. One peak at 709.8 ± 0.2 eV was associated with Fe^2+^ in octahedral sites, and the other one at 711.1 ± 0.2 eV with a broader full width at half maximum (FWHM) was assigned to Fe^3+^ in both octahedral and tetrahedral sites.^37,38^ Curve-fitting results showed that the Fe^2+^/Fe^3+^ ratio in AC-Mt, GO-Mt and Mt was 0.5, 0.49 and 0.51, respectively, close to the stoichiometry of magnetite. However, after seven consecutive cycles, the Fe^2+^/Fe^3+^ ratio of AC-Mt, GO-Mt and Mt became 0.36, 0.33 and 0.27, respectively. The decreasing Fe^2+^/Fe^3+^ ratios indicate that the surfaces of all catalysts were oxidized to different extents during heterogeneous Fenton-like reactions. Moreover, the decrease of Fe^2+^/Fe^3+^ ratio was less on supported magnetite nanocomposites than bare Mt NPs, which probably suggests the synergistic effect of carbonaceous supports and magnetite NPs in catalytic reactions.

**Fig. 8:**
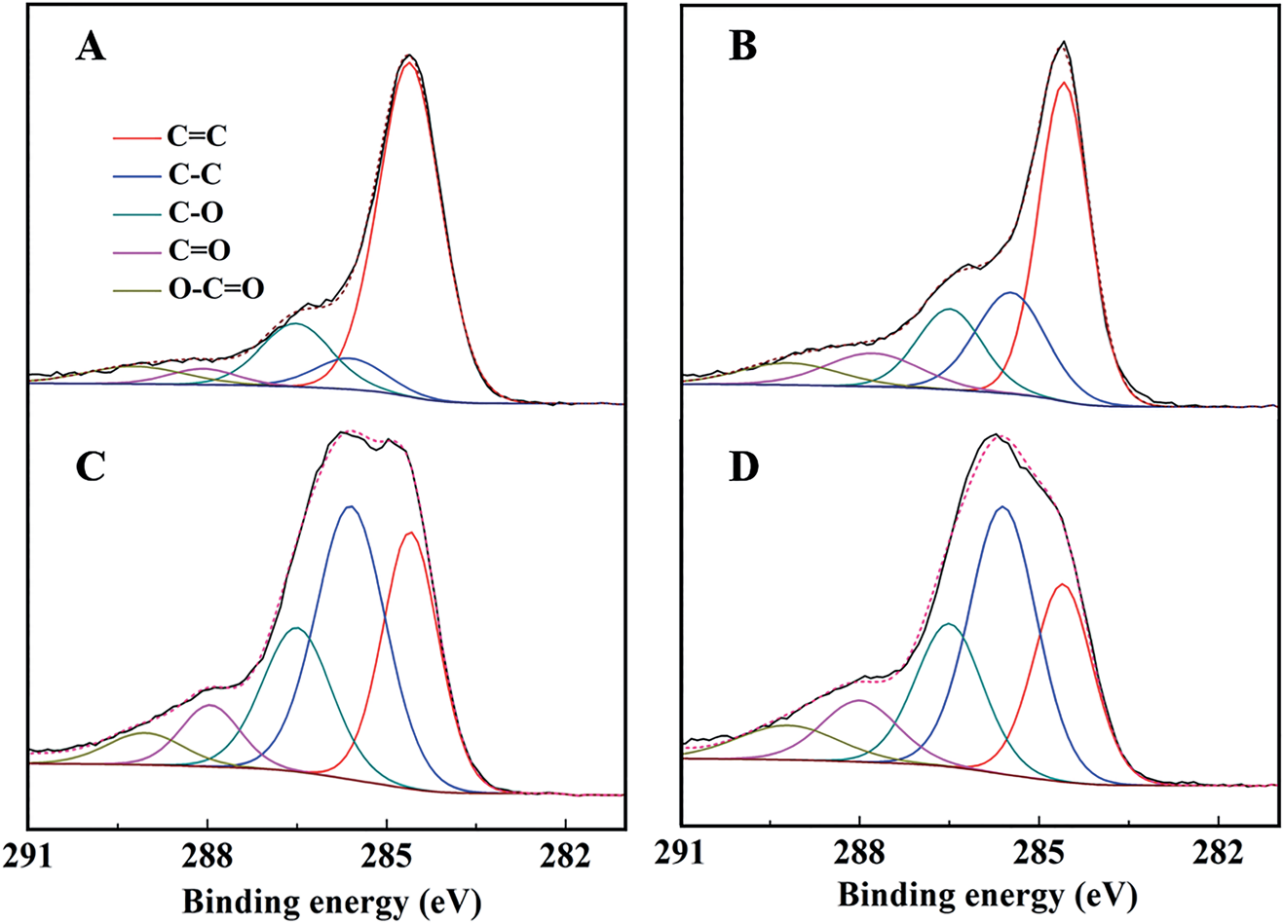
C 1s peak of AC-Mt (left) and GO-Mt (right) before (A and B) and after (C and D) seven consecutive cycles, respectively.

**Fig. 9:**
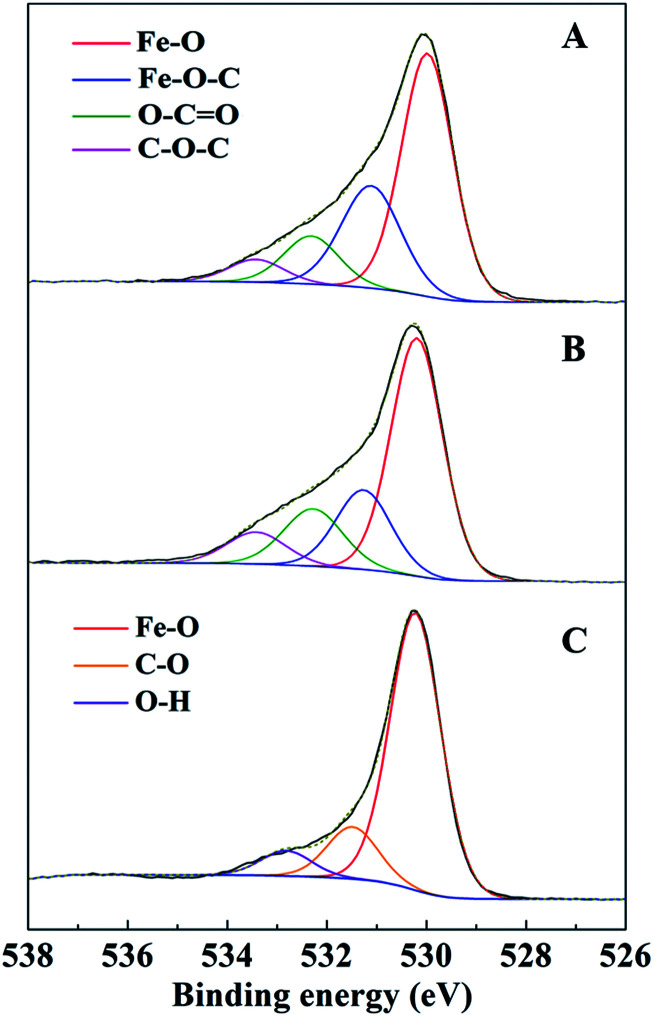
XPS analysis of O 1s peak of AC-Mt (A), GO-Mt (B) and Mt (C).

The C 1s spectra of AC-Mt and GO-Mt (Fig. 8) can be fitted into five components: C=C (284.6 eV), C–C (285.6 ± 0.1 eV), C–O (286.5 eV), C=O (288.0 ± 0.2 eV), and O–C=O (289.1 ± 0.1 eV).^12,39^ The percentage of C=C in the C 1s spectra of AC-Mt decreased from 69% to 25% after Fenton reactions (Table 2), and also that of GO-Mt decreased from 48% to 29%. Correspondingly, the percentages of C–C and C=O in the C 1s spectra of AC-Mt and GO-Mt obviously increased after Fenton reactions. These changes in the C 1s spectra probably suggest that AC or GO support in the nanocomposites was oxidized to some extent during Fenton reactions. As mentioned above, ˙OH was the dominant reactive oxygen species for MB removal. It was proposed that Fe(ii) on magnetite surface linked to the carbonaceous support might be the active sites for ˙OH production.^40^ However, the oxidation of surface Fe(ii) on magnetite was commonly accompanied with ˙OH production. The O 1s spectra of AC-Mt and GO-Mt (Fig. 9A and B) could be fitted into four components: Fe–O (530.0 ± 0.2 eV), Fe–O–C (531.2 ± 0.2 eV), COO/C=O (532.3 eV), C–O–C (533.4 eV),^4,12^ and that of bare Mt (Fig. 9C) could be fitted into three components: Fe–O (530.2 eV), C–O (531.6 eV), O–H (533.1 eV).^41^ In AC or GO supported magnetite, electrons could be transferred from the support to Fe(iii) on magnetite surface through Fe–O–C bonds and regenerate Fe(ii),^40^ which can alleviate surface deactivation and facilitate ˙OH production. Although AC and GO are both carbonaceous materials with the ability to recharge magnetite surface *via* Fe–O–C bonds, their different structures could significantly influence the number of Fe–O–C bonds formed between the support and Mt NPs. In AC-Mt, magnetite NPs were well dispersed on the AC support with a porous structure, but magnetite NPs in GO-Mt were wrapped by GO nanosheets and tended to aggregate. Thus, there were more Fe–O–C bonds formed in AC-Mt than in GO-Mt, as shown in the O 1s spectra of AC-Mt and GO-Mt (Fig. 9 and Table 3). The results indicate that immobilizing magnetite nanoparticles onto carbonaceous materials with different structures can obviously influence catalytic activity, surface deactivation, and stability of the nanocomposite catalysts in heterogeneous Fenton-like reactions.

**Table 2:** Deconvolution results of C 1s spectra for AC-Mt and GO-Mt before and after stability test

	C=C	C–C	C–O	C=O	O–C=O
Binding energy/eV	284.6	285.6 ± 0.1	286.5	288.0 ± 0.2	289.1 ± 0.1
AC-Mt	69%	6%	15%	4%	6%
AC-Mt after 7 runs	25%	38%	20%	10%	7%
GO-Mt	48%	21%	15%	9%	7%
GO-Mt after 7 runs	29%	38%	20%	8%	5%

**Table 3:** Deconvolution results of O 1s spectra for AC-Mt, GO-Mt and Mt

	Fe–O	Fe–O–C	COO; C=O	C–O–C
Binding energy/eV	530.0 ± 0.2	531.2 ± 0.2	532.3	533.4
AC-Mt	56%	27%	11%	6%
GO-Mt	56%	20%	15%	9%
	Fe–O	C–O	O–H	—
Binding energy/eV	530.2	531.6	533.1	—
Mt	61%	33%	6%	—

## 4. Conclusions

Using carbonaceous materials to prepare highly dispersed magnetite catalysts has been widely recognized, but catalytic activity of carbon-supported magnetite nanocomposites is strongly dependent on the structure of carbon supports. In this study, AC-Mt and GO-Mt with the same magnetite to support mass ratio of 1 : 1 were prepared and studied for MB removal in heterogeneous Fenton-like reactions. The porous structure of AC in AC-Mt promoted the dispersion of Mt nanoparticles, increased reactive surface area for catalytic reactions, and facilitated the diffuse of reactants and products. Moreover, electron transfer from AC to Mt nanoparticles *via* Fe–O–C bonds could alleviate surface oxidation of Mt during catalytic reactions and promote ˙OH production. However, the wrapping of GO around magnetite NPs or extensive aggregation of GO-Mt composites inhibited the diffusion of H_2_O_2_ and products between supported magnetite nanoparticles and bulk solution, resulting in slow ˙OH production and MB removal. Although GO nanosheets and AC are both carbonaceous materials with large surface area, their obviously different catalytic activities shown in this study suggest that the nanocomposite structure formed by the support and magnetite nanoparticles is also very important for catalytic behavior, besides specific properties of supports or nanoparticles. Manipulating the structure of carbon-supported magnetite nanocomposites to increase accessible reactive sites on catalysts or promote the diffusion of reactant and products onto/from catalysts could be a good way to design highly efficient Fenton catalysts.

## Conflicts of interest

There are no conflicts to declare.

## Supplementary Material

RA-008-C8RA02286K-s001
