# Supplementary material for: Structural effects on the catalytic activity of carbon-supported magnetite nanocomposites in heterogeneous Fenton-like reactions
Source: RSC Adv. 2018 May 1;8(29):16193–201. doi: 10.1039/c8ra02286k (PMC9080278; doi:10.1039/c8ra02286k)
Supplement: RA-008-C8RA02286K-s001 [file RA-008-C8RA02286K-s001.pdf]

Electronic Supplementary Material (ESI) for RSC Advances.
This journal is © The Royal Society of Chemistry 2018

Supplementary Information

Structural effects on catalytic activity of carbon-supported magnetite nanocomposites in heterogeneous Fenton-like reactions

Hongmei Zang[a], Chunyan Miao[b], Jianying Shang[c], Yingxin Liu[b], Juan Liu[a,*]

[a]The Key Laboratory of Water and Sediment Sciences, Ministry of Education, College of Environmental Sciences and Engineering, Peking University, Beijing 100871, China

[b]School of Gemmology, China University of Geosciences, Beijing, 100083, China

[c]Department of Soil and Water Sciences, China Agricultural University, Beijing 100193, China

To be submitted to ***Rsc Advances***

* Corresponding author:

J. L.

Address: College of Environmental Sciences and Engineering, Peking University, Beijing 100871, China

Email: juan.liu@pku.edu.cn

10 pages

---

3 Sections

1 Table

4 Figures

Section S1. Materials and reagents

Graphite powder, Potassium permanganate ($KMnO_4$), methylene blue trihydrate ($MB \cdot 3H_2O$), 2,4-dinitrophenylhydrazine (DNPH) were purchased from Sinopharm Chemical Reagent Co., Ltd. (Shanghai, China). $FeCl_2 \cdot 4H_2O$ and $FeCl_3$ were obtained from Xilong Chemical (Guangdong, China). Activated carbon (100-400 mesh) and dimethyl sulfoxide (DMSO) were supplied by Sigma-Aldrich. All chemicals used in this study were analytical grade or higher and used as received without further purification.

Section S2. Synthesis and characterization of GO, Mt, GO-Mt, and AC-Mt

GO was synthesized from graphite powder using a modified Hummers method. Briefly, 300mL of H_2SO_4/H_3PO_4 (90/10, v/v) was added into a mixture of 2.25g graphite and 13.5g $KMnO_4$ in a 1 L beaker. The mixture was then heated to 50℃ and continuously stirred for 12 hr. After that, 300ml of water-ice mixture with 2.25mL hydrogen peroxide (H_2O_2, 30%) were poured into the mixture. The product was collected by centrifugation at 4000 rpm for 10 minutes at room temperature. In order to remove residual reagents, the precipitate was washed with 30% hydrochloric acid solution, and then repeatedly washed with Milli-Q (> 18 MΩ cm resistivity, Millipore) water. Finally, the washed precipitates were added in Milli-Q water and sonicated (40 kHz, 300 W) for 3 hr to obtain the stock GO suspension with a solid concentration of 9.76 mg/ml.

Mt nanoparticles (NPs) were synthesized in an anoxic glovebox (N_2 atmosphere, lower than 1 ppm residual O_2) according to the co-precipitation method. 4.18 g/L $FeCl_2 \cdot 4H_2O$ and 6.89 g/L $FeCl_3$ were mixed and stirred vigorously at room temperature in a 500 mL beaker. 43.81 mL Amonium (NH_4OH) solution (28%, v/v) was added dropwise to the mixture. After that, the resulting mixture was rapidly stirred for another 30 min at room temperature. Finally, the particles were collected by magnetic separation and washed three times with degassed and deionized milli-Q water (DDW) to remove possible residual metal chlorides.

The GO-Mt or AC-Mt nanocomposites were synthesized in the anoxic glovebox similarly via the chemical co-precipitation method described above, but with the

addition of 6.7 g/L AC and 1 g/L GO, representatively. To obtain the magnetite-to-substrate ratio of 1:1, a mixture of 5.72 g/L $FeCl_2 \cdot 4H_2O$ and 9.34 g/L $FeCl_3$ was used for the synthesis of AC-Mt, and a combination of 2.58 g/L $FeCl_2 \cdot 4H_2O$ and 4.20 g/L $FeCl_3$ was used for GO-Mt synthesis. The products were also collected by magnetic separation and washed repeatedly using DDW. After washing, the synthetic nanocomposites were resuspended in DDW in serum bottles that were stored in dark inside the glovebox.

The mass ratios of magnetite to support in nanocomposites were determined by acid digestion. 0.3 mL of supported Mt nanocomposites stock suspension was dissolved in 2.7 mL of 5M HCl overnight under continuous shaking, and then filtered through a 0.22 μm syringe filter. After that, the filtrates were diluted 5,000 times with 2% nitric acid solution. Total Fe concentration in the diluted filtrates was determined with an inductively coupled plasma optical emission spectrometry (Prodigy High Dispersion ICP-OES, Teledyne Leeman Laboratories, Hudson, NH). The equivalent concentration of magnetite in all suspensions was calculated from the measured Fe concentration according to the ideal stoichiometry of magnetite (Fe_3O_4). In addition, the weight concentration of particles in the stock suspensions was measured from the difference in the weights of 3 mL suspension before and after drying. All concentration measurements were performed in triplicates.

Section S3. Determination of •OH in heterogeneous Fenton-like reaction

Briefly, 250 mM DMSO, as the scavenger of •OH, was added to the reactor after the catalyst was equilibrated with the HCl solution. Then, H_2O_2 was added to initiate the Fenton-like reaction. At desired time points, an aliquot of 2 mL samples was taken out and filtered by 0.22 μm syringe filter. Then, the filtrate was added into 2.5 mL pH 4.0 H_3PO_4-NaH_2PO_4 buffer solution and 0.2 mL of 6 mM DNPH solution. The mixture was diluted to 5 mL with DDW and maintained at room temperature for 30 min. Then, the concentrations of DNPH and the corresponding hydrazine (HCHO-DNPH) in the samples were measured by High Performance Liquid Chromatography (HPLC, Agilent Technologies 2004, G2171BA).

Table S1. Brunauer-Emmett-Teller (BET) specific surface area results of bare AC, AC-Mt and Mt.

**Materials**	**AC**	**AC-Mt**	**Mt**
**SA (m^2/g)**	962.0	512.1	108.0

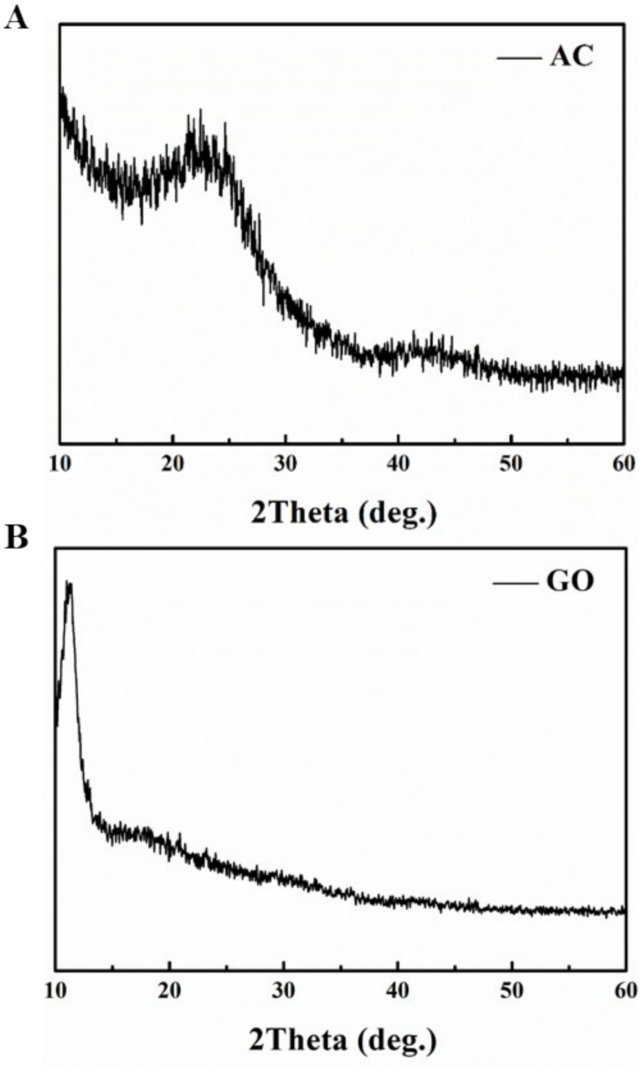

Fig.S1 XRD patterns of bare AC (A) and GO (B).

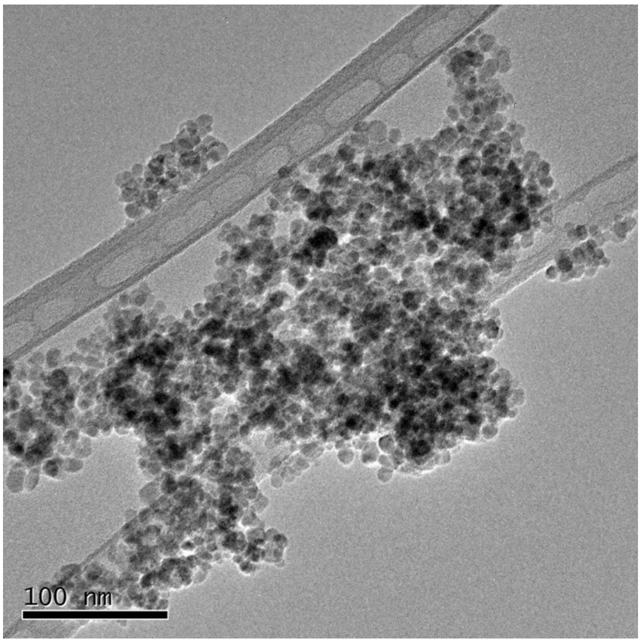

Fig. S2 Bright-field TEM image of bare Mt nanoparticles.

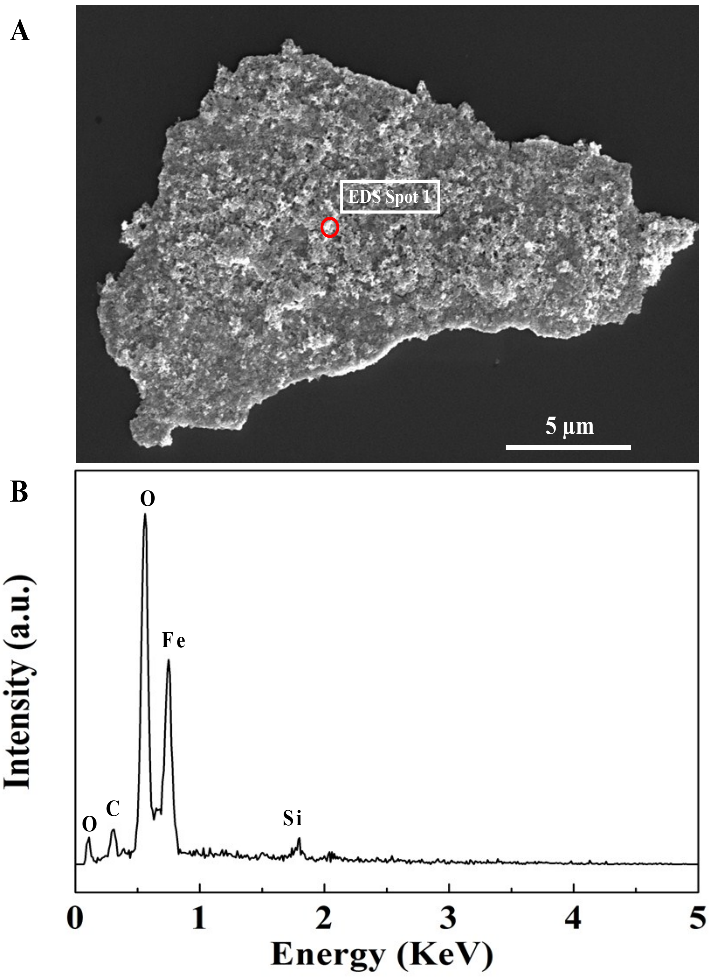

Fig.S3 SEM image (A) of AC-Mt and the EDS spectrum (B) of the bright aggregates (denoted by the red circle in Fig. S3A) on AC-Mt. The signal of Si is from the silicon chip support that was used to load the sample. Scale bar is 5 μm.

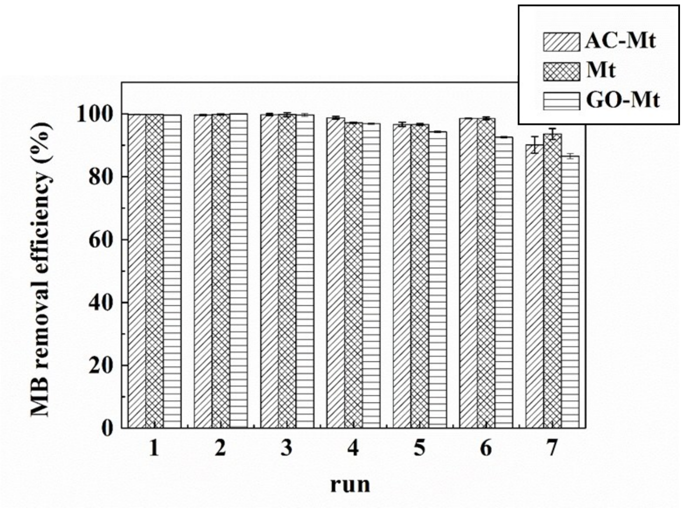

Fig.S4 Stability test of MB removal using AC-Mt, Mt and GO-Mt in heterogeneous Fenton-like systems.
